# The diagnostic dilemma of a multilocular renal cyst: a case report

**DOI:** 10.1186/1752-1947-3-79

**Published:** 2009-10-15

**Authors:** Gaural Patel, Muhammad Choudhry, Kokila Lakhoo

**Affiliations:** 1 Children's Hospital, Oxford and University of Oxford, Headley Way, Oxford, OX3 9DU, UK

## Abstract

**Introduction:**

Renal cysts presenting in childhood are rare. Historically, renal cysts have been subject to variable nomenclature which has contributed to diagnostic difficulties. They can occur as solitary, unilateral or multiple lesions. Cysts can be further classified according to loculation, communications within the structure and tissue types present.

**Case presentation:**

We report the case of a 15-month-old Caucasian boy presenting with abdominal distension as the only clinical symptom. On examination, an ill-defined abdominal mass was found. This was confirmed on ultrasound to be a multiseptated cystic mass with a solid element, arising from the right side of the abdomen. Despite further imaging, the origin of the mass could not be identified. The mass was suspected of malignancy but all blood tests and tumour markers were normal. The mass proved to be a diagnostic challenge. The renal origin of the mass was only confirmed at surgery.

**Conclusion:**

Imaging appears to be unreliable in differentiating benign cysts from malignant renal tumours, raising a diagnostic dilemma where surgery seems the only way to reliably establish aetiology of the mass.

## Introduction

A multilocular cystic lesion of the kidney presents a diagnostic dilemma. Using current diagnostic techniques, it is extremely difficult to differentiate renal neoplasms from essentially benign conditions with little malignant potential.

This condition has been reported in the literature under many different names. Fifteen years ago, a literature review highlighted the various synonyms given to this condition, including cystic nephroma, cystic partially differentiated nephroblastoma, polycystic nephroblastoma, multilocular cystic nephroma, unilateral polycystic kidney, cystic adenoma and papillary cystadenoma [1].

We report a case of a multilocular renal cyst and a recent literature summary.

## Case presentation

A 15-month-old Caucasian boy presented with a history of progressive abdominal distension for a 6-week duration. He was experiencing no renal or gastrointestinal symptoms and his routine antenatal scans at 20 weeks gestation were reported as normal. Physical examination showed him to be unwell, with a pulse rate of 150 beats/minute and blood pressure of 108 per 82 mmHg. He had a largely distended and tense abdomen, with general discomfort on palpation but no signs of peritonitis. There was, however, an ill-defined cystic mass palpable in the right side of his abdomen.

**Figure 1:**
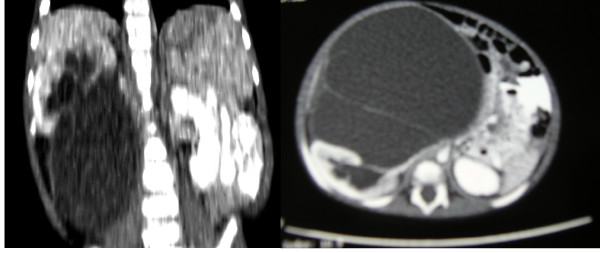
**Axial/coronal computed tomographic scan of abdomen showing cystic, loculated mass inferior to right kidney**.

No abnormalities were evident from routine blood tests (haematology and coagulation, biochemistry, C-reactive protein and liver function tests). Further laboratory measurements of urinary electrolytes, alpha-feto protein and beta human chorionic gonadotropin levels were also all within normal limits. An abdominal ultrasound scan showed a large, cystic, multiseptated mass containing a small solid nodule, which was suspected of malignancy. The lesion was located in his right flank which extended across the midline and inferiorly into the pelvis. The origin of this mass could not be identified. A chest, abdominal and pelvic computed tomographic scan was performed, that showed a 14.7 × 13.6 × 15.2 cm complex septated cystic mass arising from the peritoneum, containing enhancing septae and solid nodules (Figure 1). The right kidney was seen to be mildly hydronephrotic and displaced supero-laterally but appeared separate from the mass. The left kidney was reported normal in size and texture. No intrathoracic and abdominal lymphadenopathy was reported. The overall impression was of a teratoma with a differential of mesenchymal hamartoma or a complicated mesenteric cyst.

On day 6 of his admission, an exploratory laparotomy was performed due to increasing pain and distress suffered by the patient. The operative finding was a large cyst arising from the lower pole of the right kidney which was adherent to the duodenum, caecal mesentery, and colonic mesentery. The cyst was completely excised and a right nephro-ureterectomy was also carried out. The histology of the mass was reported as originating from the kidney with segmental dysplasia and haemorrhagic cyst formation, suggesting a benign multilocular renal cyst. The postoperative course was uneventful, and the patient is well at 5 years of age.

## Discussion

The first description of a multilocular renal cyst was made in 1892 by Edmunds, who called it a "cystic adenoma of the kidney". It was not until 1951 that Powell suggested further diagnostic criteria, which included site, complexity and association with renal tissue [2].

These criteria were later modified by Boggs and Kimmelstiel in 1956 to include the presence of immature tissue in the intervening septa as a feature of the cyst [3]. The condition is rare with a reported incidence of 2.4% of all primary renal lesions [4,5].

The condition seems to present predominantly among Caucasian children, with males affected twice as often as females. Conversely, the ratio increases eight-fold among females who develop the condition during adulthood [1].

In the last 10 years only two cases of this condition have been reported, with no discernable diagnostic improvement. This is suggestive of a trend, though not conclusive.

A multilocular renal cyst usually presents with a painless abdominal mass in the majority of cases, but it can also present less often with abdominal or flank pain, haematuria, hypertension or as an incidental finding [1]. There are no conclusively proven genetic associations.

Imaging is useful in identifying a mass. Diagnostic difficulties, however, have been reported, as noted in our case. Computed tomography and ultrasound scans may identify a complete cyst but are unable to specify the origin of the lesion in all cases [6].

## Conclusion

There have been no reported cases of malignant behaviour or potential in multilocular renal cysts [1,7,8]. Imaging, percutaneous tissue biopsy and cyst aspiration cannot differentiate a multilocular cyst from multicystic renal cell carcinoma, adenocarcinoma, or Wilms' tumour [1,9,10]. Thus the management of this lesion, for diagnostic accuracy as well as for treatment, is either by total nephrectomy or partial nephron sparing surgery [1,7,8]. Metachronic lesions have been reported in the literature and should be explored on imaging. It is recommended that a close follow up on the patient be performed using clinical evaluation and the best imaging techniques available [7]. In the future, better diagnostic methods to identify multilocular renal cysts that are essentially benign may spare the kidney.

## Competing interests

The authors declare that they have no competing interests.

## Authors' contributions

GP drafted the manuscript of this case report. MC provided the literature review. KL contributed to the conceptualization, review, data preparation, analysis, interpretation and coordination of this study. All authors read and approved the final manuscript.

## Consent

Written informed consent was obtained from the patient's parents for the publication of this case report and any accompanying images. A copy of the written consent is available for review by the Editor-in-Chief of this journal.

## References

[B1] Castillo OA Boyle ET Kramer SA Multilocular cysts of kidney: A study of 29 patients and review of literature Peditr Urol 1991 37 156 162 10.1016/0090-4295(91)80214-R 1846992

[B2] Powell T Shackman R Johnson HD Multilocular cysts of the kidney Br J Urol 1951 23 142 10.1111/j.1464-410X.1951.tb02576.x 14839238

[B3] Boggs LK Kimmelstiel P Benign multilocular cystic nephroma: report of two cases of so called multilocular cyst of the kidney J Urol 1956 76 530 541 13377464 10.1016/S0022-5347(17)66732-6

[B4] Parienty RA Computed tomography of multilocular cystic nephroma Radiology 1981 140 135 139 7244215 10.1148/radiology.140.1.7244215

[B5] Gallo GE Penchansky L Cystic nehroma Cancer 1977 39 1322 1327 10.1002/1097-0142(197703)39:3<1322::AID-CNCR2820390346>3.0.CO;2-L 199349

[B6] Silverman SG Mortele KJ Tuncali K Jinzaki M Cibas ES Hyperattenuating renal masses: etiologies, pathogenesis, and imaging evaluation Radiographics 2007 27 4 1131 1143 10.1148/rg.274065147 17620471

[B7] Ferrer FA McKenna PH Partial nephrectomy in a metachronous multilocular cyst of the kidney (cystic nephroma) J Urol 1994 151 1358 1360 8158788 10.1016/s0022-5347(17)35258-8

[B8] Okada T Yoshida H Matsunaga T Kouchi K Ohtsuka Y Saitou T Horie H Ohnuma N Nephron-sparing surgery for multilocular cyst of the kidney in a child J Pediatr Surg 2003 38 1689 1692 10.1016/S0022-3468(03)00589-X 14614728

[B9] Takeuchi T Tanaka T Tokuyama H Kuriyama M Nishiura T Multilocular cystic adenocarcinoma: a case report and review of the literature J Surg Oncol 1984 25 136 140 10.1002/jso.2930250217 6694402

[B10] Laperriere J Filion R Houde M Charghi A Renal cell carcinoma presenting as multilocular cystic mass Urology 1986 28 155 158 10.1016/0090-4295(86)90111-1 3739123

